# Supplementary material for: 18F-FDG and 11C-Methionine PET/CT in Newly Diagnosed Multiple Myeloma Patients: Comparison of Volume-Based PET Biomarkers
Source: Cancers (Basel). 2020 Apr 23;12(4):1042. doi: 10.3390/cancers12041042 (PMC7226577; doi:10.3390/cancers12041042)
Supplement: Supplementary file 1 [file cancers-12-01042-s001.pdf]

Supplementary Materials

^{18}F-FDG and ^{11}C-Methionine PET/CT in Newly Diagnosed Multiple Myeloma Patients: Comparison of Volume-Based PET Biomarkers

Maria I Morales-Lozano, Oliver Viering, Samuel Samnick, Paula Rodriguez-Otero, Andreas K Buck, Maria Marcos-Jubilar, Leo Rasche, Elena Prieto, K Martin Kortüm, Jesus San-Miguel, Maria J. Garcia-Velloso and Constantin Lapa

Table S1. Patients' characteristics.

No.	M component (g/l)	FLC (mg/l)	BM Involvement (%)	LDH	Albumin	Creatinine	Haemoglobin	Platelet count	Calcium	CRP	B2M
1	4	4440	40	185	4,3	2,1	12	130	2,2	0,7	5,5
2	n/a	233	30	187	4,1	0,7	12	299	2,5	0,2	2
3	41,3	12	65	126	4,1	1,2	12	266	2,5	0,2	5,1
4	n/a	345	25	219	3,2	1	12	810	2,5	2,8	4,5
5	n/a	107	1	193	4,7	0,9	13	193	2,4	0,2	2,6
6	50	48	90	102	3,6	0,8	11	292	2,5	n/a	2,7
7	34	337	n/a	172	4,3	2,2	11	139	2,4	0,7	17,8
8	28	254	15	177	4,4	0,7	15	192	2,4	0,1	1,8
9	2	17020	75	194	5,1	1	11	104	2,3	0	5,5
10	n/a	905	90	720	4,6	1,1	13	272	2,4	0,3	2,2
11	30	551	45	168	3,8	0,9	13	277	2,3	0,1	1,9
12	34	5297	90	185	3,8	1,2	10	207	2,1	0,9	9,6
13	n/a	2947	20	387	4,3	0,9	8	283	2,5	3,6	3,2
14	n/a	495	50	181	3,6	0,9	14	229	2,2	0,1	1,6
15	15	2971	30	153	4,3	0,9	12	193	2,2	1,5	2,4
16	2,5	158	20	185	4,3	0,5	12	227	2,2	0	1,7
17	1,9	291	8	498	4,5	0,7	16	240	2,2	3,2	1,8
18	1,9	n/a	8	n/a	4,2	0,8	14	n/a	n/a	n/a	1,7
19	2,4	216	44	272	4,4	1,1	20	140	2,4	1,5	3,8
20	4,6	64	39	101	4	0,7	12	182	2,1	0,1	2,3
21	0	40	21	134	3,3	0,9	15	225	n/a	n/a	2,4
22	7	76	34	153	2,5	1,3	7	106	8,8	24	6,8

*n/a = information not available.

Table S2. Thresholds selected for FIJI software.

No.	Pattern ^{18}F-FDG	Selected Threshold ^{18}F-FDG	Pattern ^{11}C-MET	Selected Threshold ^{11}C-MET
1	focal	SUV>4	focal	SUV>4
2	focal	SUV>4	combined	SUV > 41% SUVmax
3	diffuse	SUV > 41% SUVmax	diffuse	SUV > 41% SUVmax
4	diffuse	SUV > 50% SUVmax	combined	SUV > 30% SUVmax
5	focal	SUV > 41% SUVmax	combined	SUV > 41% SUVmax
6	focal	SUV > 41% SUVmax	combined	SUV > 41% SUVmax
7	diffuse	SUV > 50% SUVmax	diffuse	SUV > 41% SUVmax
8	focal	SUV > 41% SUVmax	combined	SUV > 41% SUVmax
9	diffuse	SUV > 41% SUVmax	diffuse	SUV > 50% SUVmax
10	combined	SUV > 41% SUVmax	combined	SUV>4
11	focal	SUV > 1	combined	SUV > 41% SUVmax
12	combined	SUV > 41% SUVmax	combined	SUV > 41% SUVmax
13	focal	SUV>4	combined	SUV > 41% SUVmax
14	combined	SUV > 41% SUVmax	combined	SUV > 41% SUVmax
15	diffuse	SUV > 41% SUVmax	diffuse	SUV > 41% SUVmax
16	focal	SUV>4	focal	SUV>4
17	diffuse	SUV > 41% SUVmax	diffuse	SUV > 41% SUVmax
18	focal	SUV > 41% SUVmax	focal	SUV > 41% SUVmax
19	focal	SUV > 41% SUVmax	combined	SUV>4
20	combined	SUV > 41% SUVmax	combined	SUV > 41% SUVmax
21	combined	SUV > 41% SUVmax	combined	SUV > 41% SUVmax
22	combined	SUV > 41% SUVmax	diffuse	SUV > 41% SUVmax

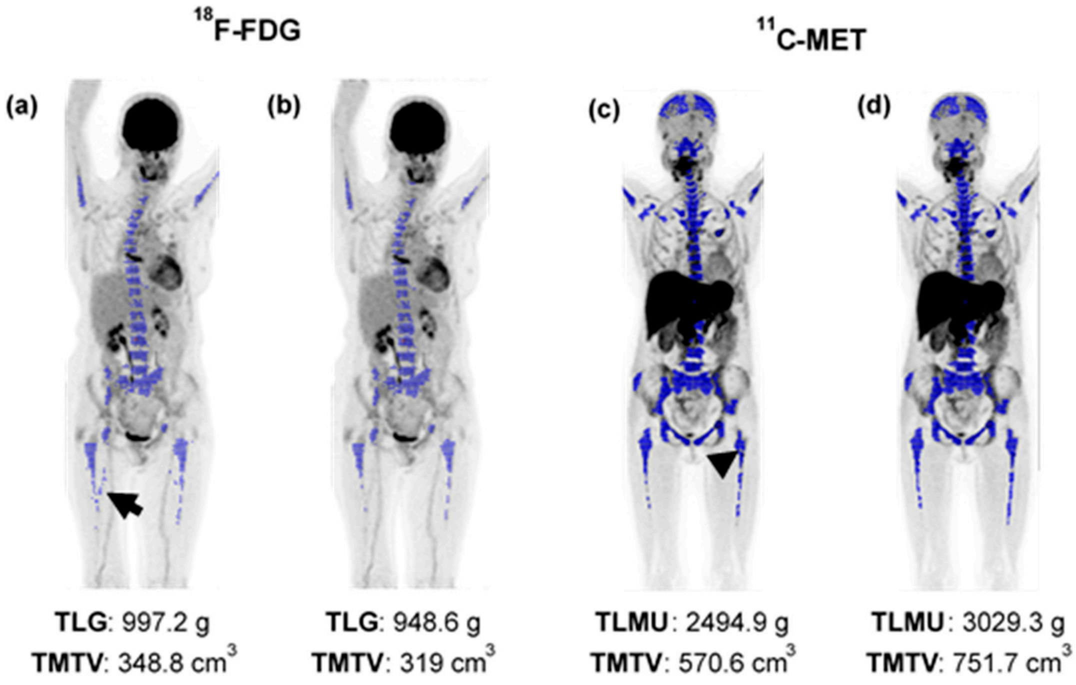

Figure S1. Example of different cut-off values performed for both tracers. A 56-year-old female with Multiple Myeloma R-ISS II with diffuse BM infiltration on ^{18}F-FDG PET/CT and a combined pattern on ^{11}C-MET PET/CT with < 3 FL. In ^{18}F-FDG PET/CT, SUV > 50% of SUVmax (**b**) was selected instead of SUV > 41% (**a**) because of slight overestimation (arrow) whereas in ^{11}C-MET, a threshold of SUV > 41% of SUVmax (**c**) underestimated the disease extent (arrow head) so the SUV > 30% of SUVmax threshold was chosen instead (**d**).

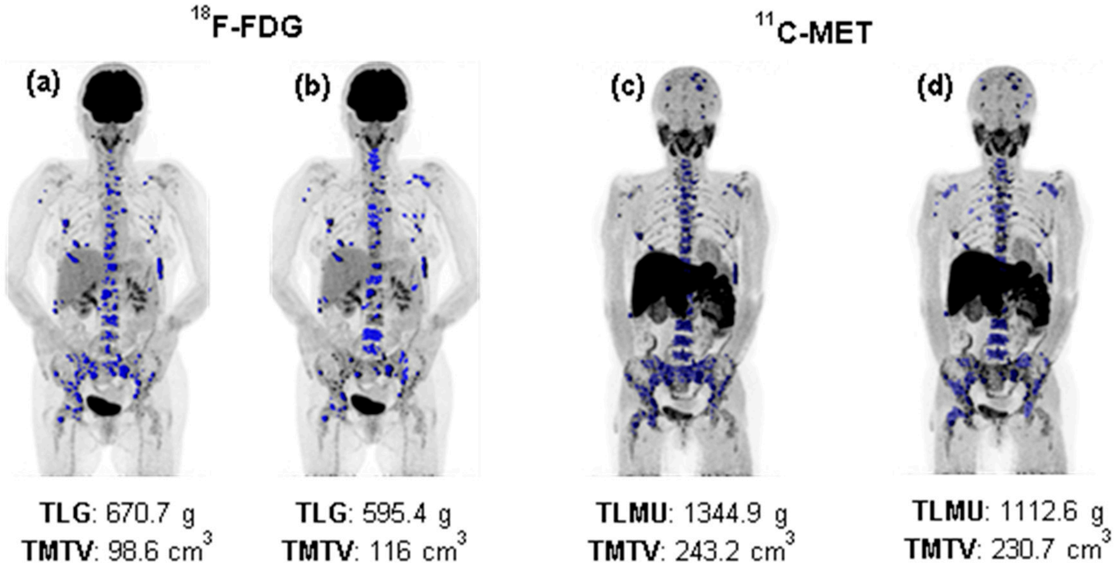

Figure S2. Example of impaired results of a fixed threshold of SUV > 41% SUVmax. Patient (patient #16) with a history of IgG kappa MM R-ISS II. This case illustrates the impaired results of selecting a fixed threshold of SUV > 41% SUVmax (**b,d**) in case of focal pattern on FDG PET/CT. Please note the multiple pelvic lesions not included when SUV > 41% of SUVmax was employed. In this case, the selected threshold for both tracers was SUV > 4 (**a,c**). Only the proximal third of femurs was affected so the distal portion is not included in the present figure.

© 2020 by the authors. Licensee MDPI, Basel, Switzerland. This article is an open access article distributed under the terms and conditions of the Creative Commons Attribution (CC BY) license (http://creativecommons.org/licenses/by/4.0/).
